# Supplementary material for: Structural insights into perilipin 3 membrane association in response to diacylglycerol accumulation
Source: Nat Commun. 2023 Jun 2;14:3204. doi: 10.1038/s41467-023-38725-w (PMC10238389; doi:10.1038/s41467-023-38725-w)
Supplement: Supplementary file 1 — Supplementary Information [file 41467_2023_38725_MOESM1_ESM.pdf]

Supplementary Information

Structural insights into perilipin 3 membrane association in response to diacylglycerol accumulation

Choi et al

Supplementary Figures

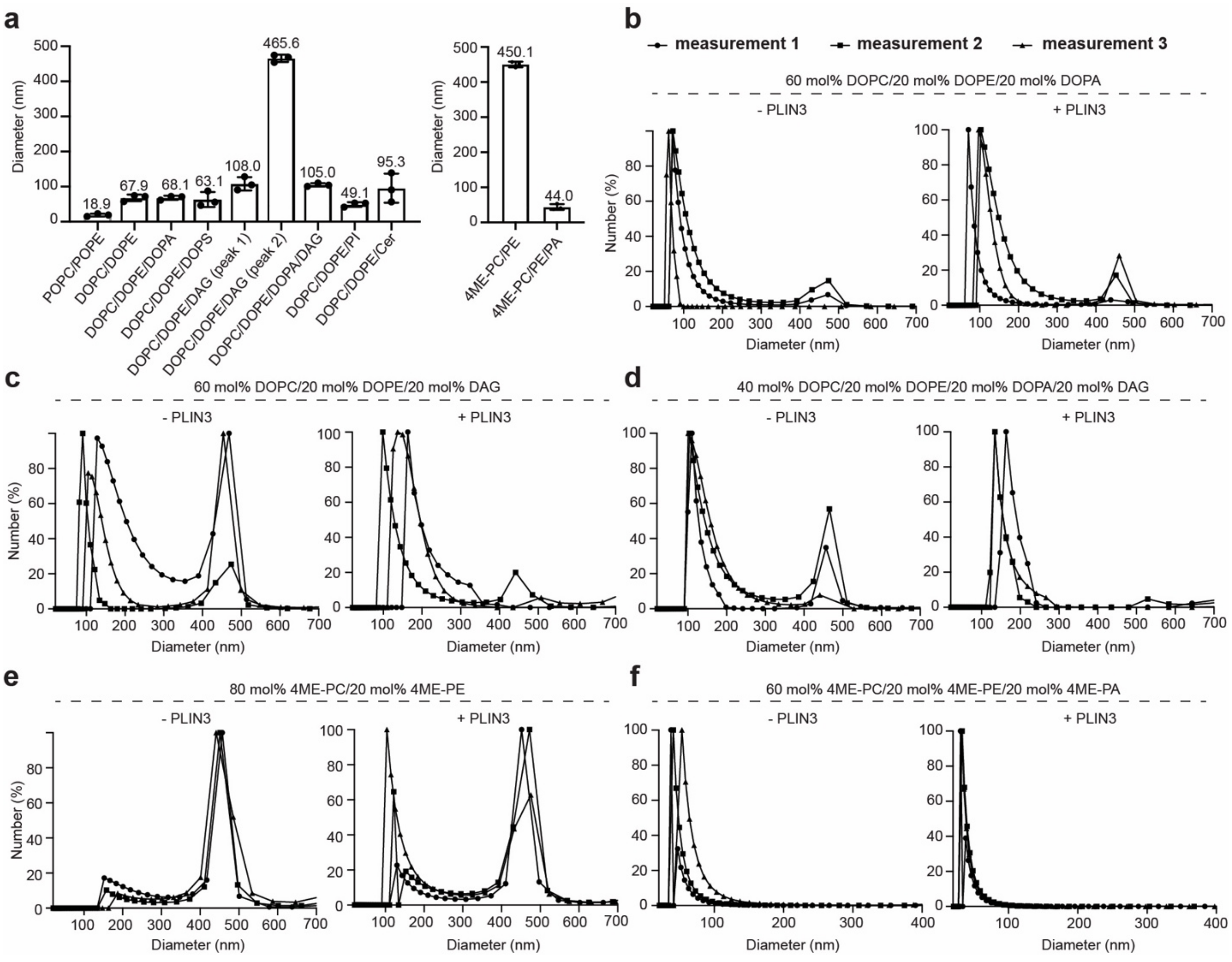

Supplementary Fig. 1. Liposome characterization by dynamic light scattering.

a) DLS analysis of liposomes with different lipid composition. Data are presented as mean values +/- SD (n=3).
b-f) DLS analysis of different liposomes with or without full length PLIN3. For each liposome, the number average size distribution (%) was recorded and plotted in the presence and absence of full length PLIN3 (n=3).
Source data are provided as a Source Data file.

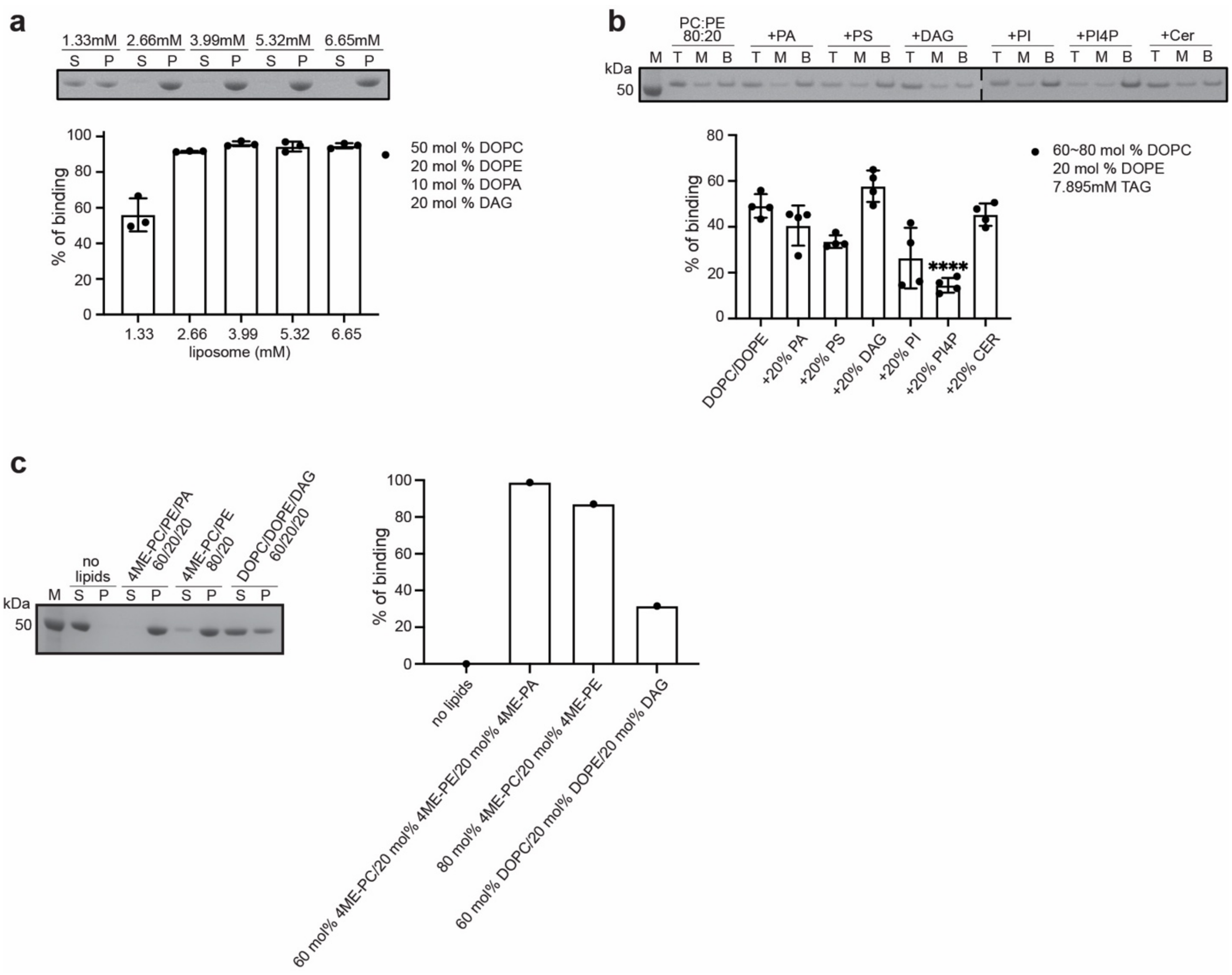

Supplementary Fig. 2. Effect of liposome concentration, lipid composition, and buffer conditions on perilipin 3 binding to liposomes and artificial lipid droplets.

a) SDS-PAGE and quantitative analysis of human PLIN3 recruitment by increasing the total amount of liposomes. A molar ratio of DOPC/DOPE/DOPA/DAG (50:20:10:20) was kept constant. Lane S and P represent unbound and bound human PLIN3 from the supernatant and pellet. Increasing liposome amount results in almost 100% recruitment of PLIN3 to liposomes. Data are presented as mean values +/- SD from three independent experiments (n=3).
b) SDS-PAGE and quantitative analysis of human PLIN3 recruitment to ALDs generated with DO-phospholipids and TAG by adding additional with 20mol% of lipids such as PS, PA, DAG, PI, PI4P and ceramide. Top, middle and bottom fractions after sucrose-gradient centrifuge were indicated as T, M and B, respectively. Lane M represents the protein ladder marker. Statistical analysis was performed using ordinary one-way ANOVA with Tukey's multiple comparisons test (n=4, ****, $p < 0.0001$).
c) SDS-PAGE and quantitative analysis of human PLIN3 recruitment to liposomes in buffer containing 100mM NaCl and 20mM HEPES pH 7.0 (n=1). Three different liposomes, 4ME-PC/4ME-PE/4ME-PA, 4ME-PC/4ME-PE and DOPC/DOPE/DAG were generated. Lane S represents unbound human PLIN3 from supernatant. Lane P represents pelleted human PLIN3 that bound to liposomes.
Source data are provided as a Source Data file.

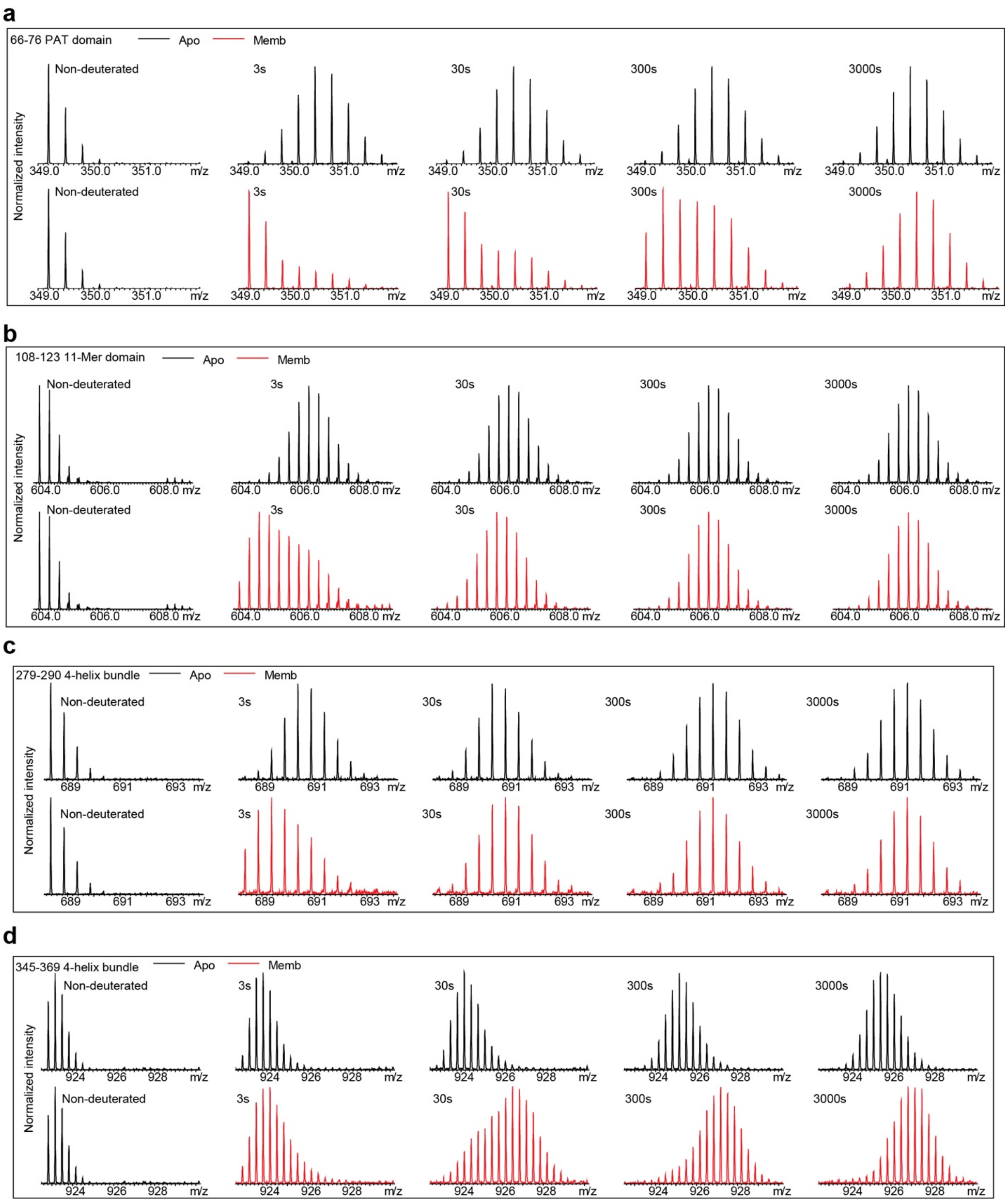

Supplementary Fig. 3. Bimodal distribution mass spectra after deuterium exchange.

a-d) Representative bimodal distribution mass spectra from the peptides at PAT domain, 11-mer repeats, and 4-helix bundle of PLIN3 after deuterium exchange.

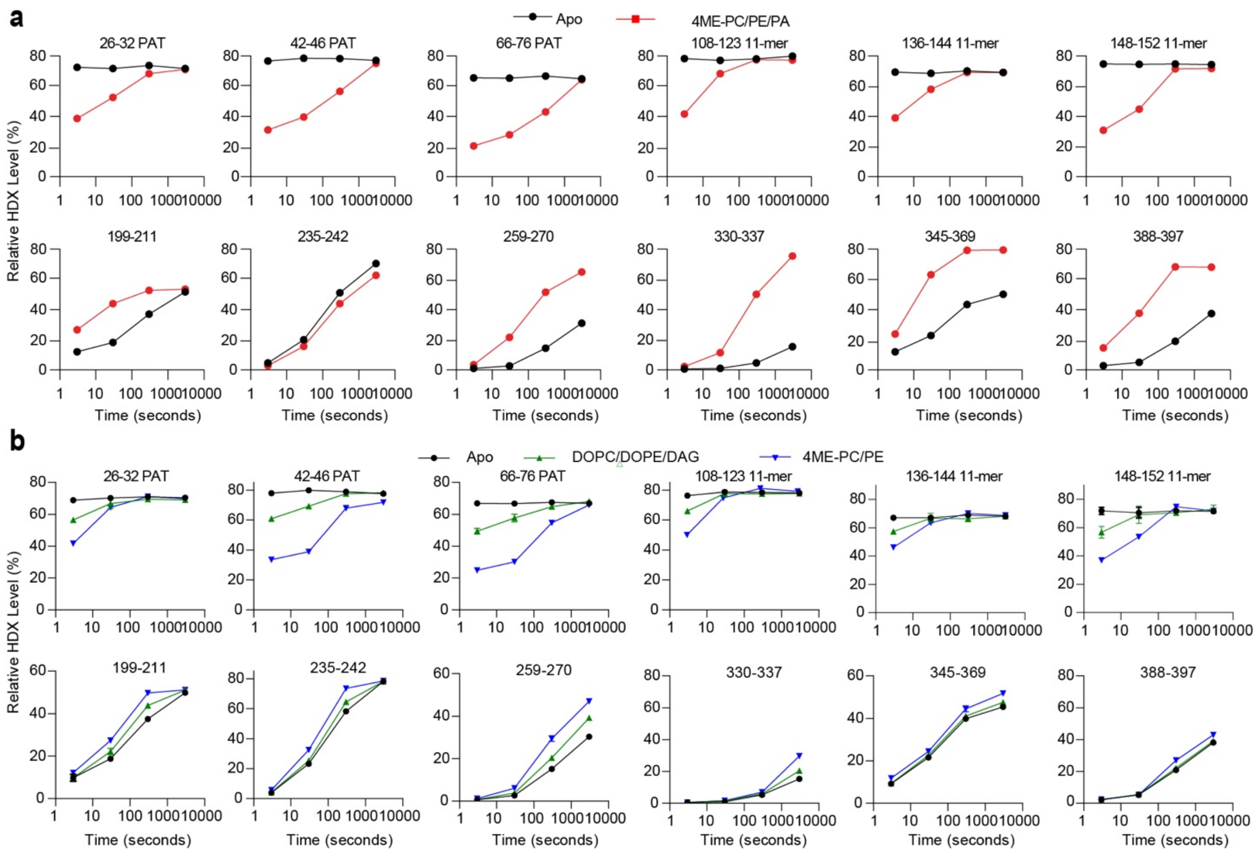

Supplementary Fig. 4. Deuterium exchange uptake plots.

a) Deuterium exchange uptake plots for selected peptides in the PAT domain, 11-mer repeats and 4-helix bundle were plotted across all timepoints as 3s, 30s, 300s and 3000s. Data are presented as mean values +/- SD from three independent experiments (n=3). Most error bars are smaller than the size of the point. Data points in the absence and presence of liposomes were colored in black and red, respectively. Liposomes were generated with 60 mol% 4ME-PC, 20 mol% 4ME-PE and 20 mol% 4ME-PA). All peptides are shown in the Source Data file.
b) Deuterium exchange uptake plots for selected peptides in the presence of two different liposomes are plotted across all timepoints as 3s, 30s, 300s and 3000s colored according the the legend. All peptides are shown in the source data. Data are presented as mean values +/- SD from three independent experiments (n=3). Most error bars are smaller than the size of the point. Source data are provided as a Source Data file.

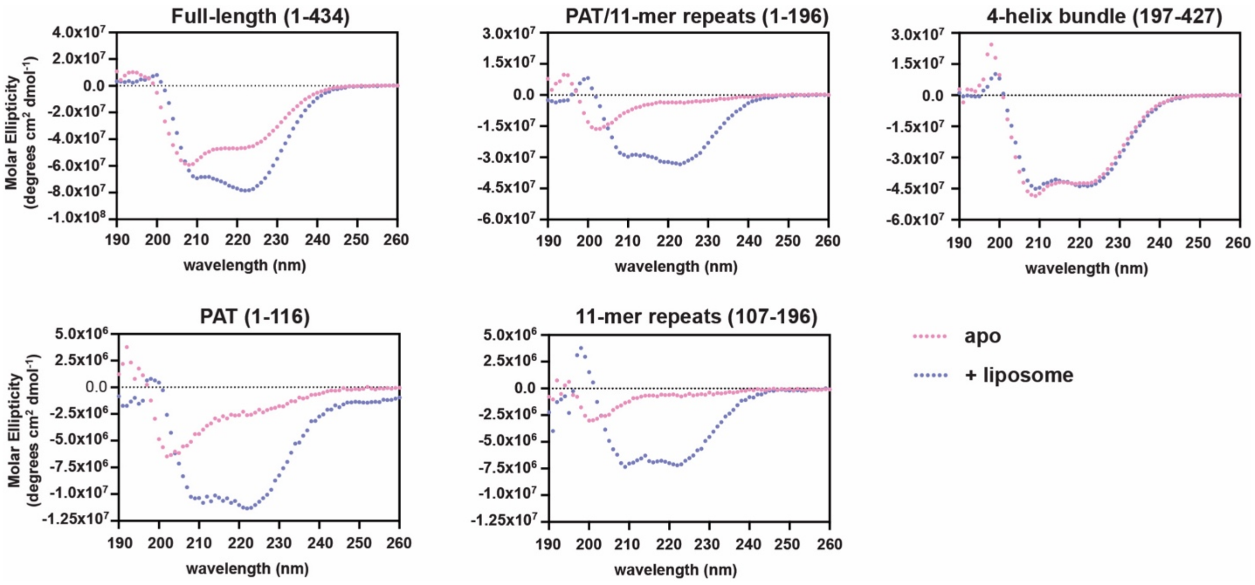

Supplementary Fig. 5. Circular dichroism analysis of secondary structure changes in perilipin 3 in the presence of liposomes.

Circular dichroism (CD) analysis of PLIN3 full length and various fragments with or without 4ME-PC/PE/PA liposomes. Source data are provided as a Source Data file.

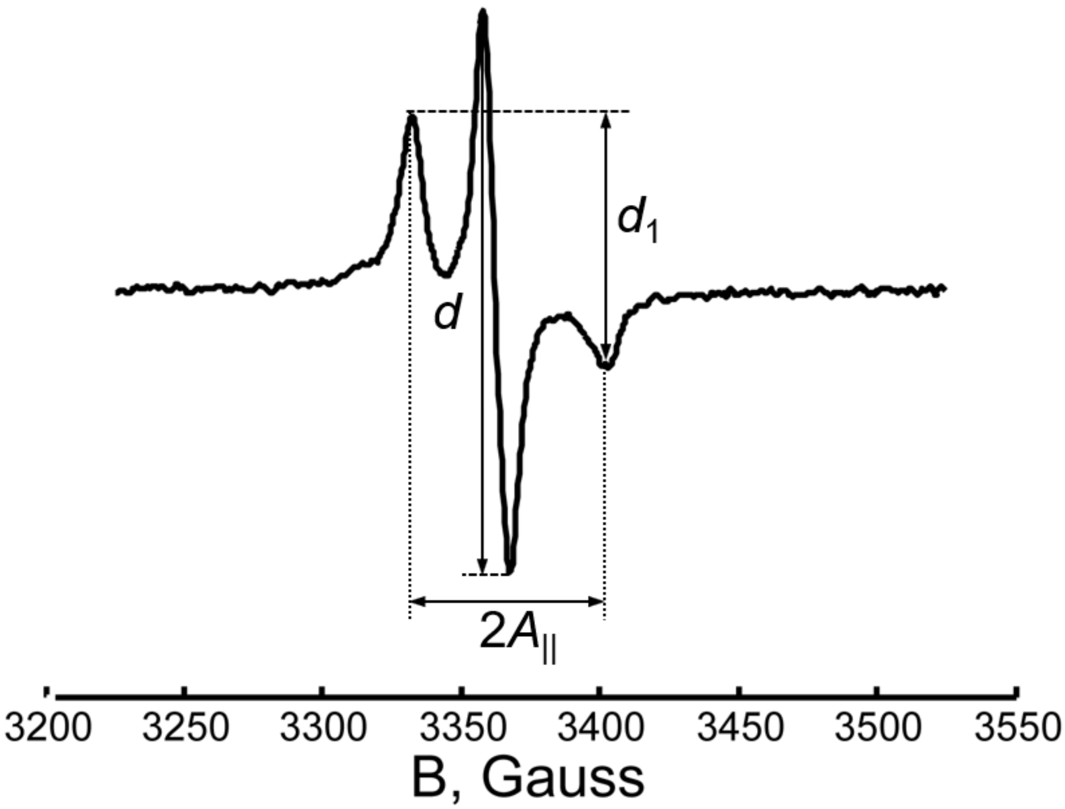

Supplementary Fig. 6. Continuous wave ESR spectrum.

CW ESR spectrum of 37C/144C in lipid recorded at 9,434 GHz frequency. The Parameter ∆, defined [1] as the ratio of d1/d, is 0.45, which for the MTSL spin label indicates the distance in range of 1.5-2.0 nm [1, 2], i.e. somewhat shorter than reported by DEER which has reduced sensitivity to distances below 2.0 nm.

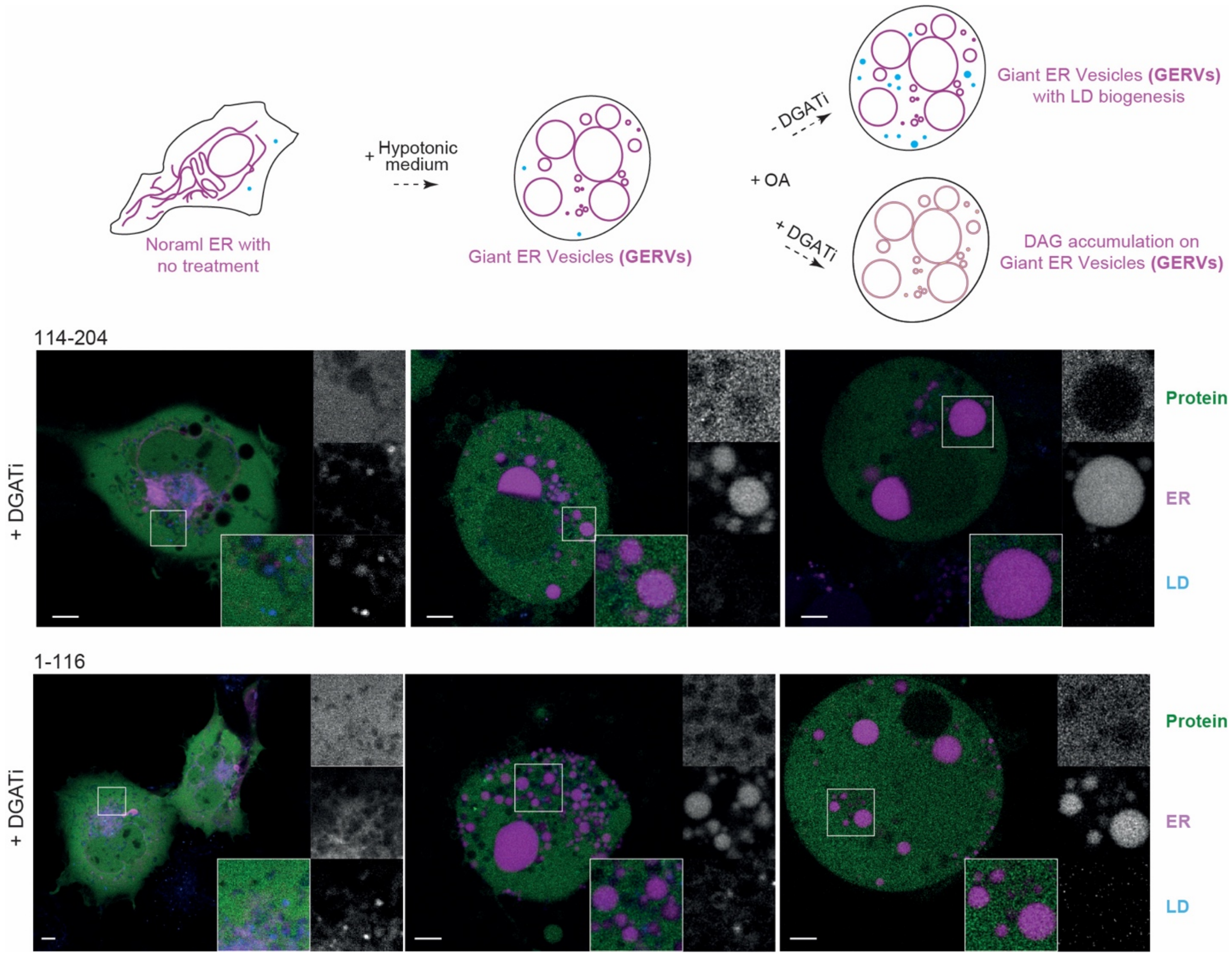

Supplementary Fig. 7. Subcellular localization of PAT domain and 11mer repeat constructs.

Subcellular localization of GFP tagged 114-204 and 1-116 constructs of PLIN3 was visualized in green in Cos7 cells under fluorescent microscope ZEISS LSM800 Airyscan. ER was visualized with ER specific marker RFP-KDEL in magenta. Lipid droplets were labeled with LipidTox Deepred to stain neutral lipids in cyan. After treated with hypotonic medium, cells were supplemented with oleate in the presence or absence of DGAT1/2 inhibitors.

Supplementary Table 1.

Summary of all HDX-MS data processing

Data set	PLIN3 order disorder Expt-1	PLIN3 Apo Expt-2	PLIN3 + 60% 4ME-PC, 20% 4ME-PE, 20% 4ME-PA liposomes Expt-2	PLIN3 Apo Expt-3	PLIN3 + 60% DOPC, 20% DOPE, 20% DAG liposomes Expt-3	PLIN3+ + 80% 4ME-PC, 20% 4ME-PE liposomes Expt-3
HDX reaction details	%D2O=84.9 % pH(read)=7.5 Temp=20ºC	%D2O=63% pH(read)=8.0 Temp=20ºC	%D2O=63% pH(read)=8.0 Temp=20ºC	%D2O=72% pH(read)=8.0 Temp=20ºC	%D2O=72% pH(read)=8.0 Temp=20ºC	%D2O=72% pH(read)=8.0 Temp=20ºC
HDX time course (seconds)	0.3s, fully deuterated	3s, 30s, 300s, 3000s	3s, 30s, 300s, 3000s	3s, 30s, 300s, 3000s	3s, 30s, 300s, 3000s	3s, 30s, 300s, 3000s
HDX controls	FD	N/A	N/A	N/A	N/A	N/A
Back-exchange	Corrected by fully deuterated sample	No correction, deuterium levels are relative	No correction, deuterium levels are relative	No correction, deuterium levels are relative	No correction, deuterium levels are relative	No correction, deuterium levels are relative
Number of peptides	144	90	90	90	90	90
Sequence coverage	99.8%	91.7%	91.7%	91.7%	91.7%	91.7%
Average peptide /redundancy	Length= 17.3 Redundancy = 5.4	Length= 13.0 Redundancy = 2.6	Length= 13.0 Redundancy = 2.6	Length= 13.0 Redundancy = 2.6	Length= 13.0 Redundancy = 2.6	Length= 13.0 Redundancy= 2.6
Replicates	3	3	3	3	3	3
Repeatability	Average StDev=0.4%	Average StDev=0.6%	Average StDev=0.6%	Average StDev=0.8%	Average StDev=1.0%	Average StDev=0.9%
Significant differences in HDX	N/A	>5% and >0.4 Da and unpaired t-test ≤0.01	>5% and >0.4 Da and unpaired t-test ≤0.01	>5% and >0.4 Da and unpaired t-test ≤0.01	>5% and >0.4 Da and unpaired t-test ≤0.01	>5% and >0.4 Da and unpaired t-test ≤0.01

Supplementary Notes

Codon optimized DNA sequence of human perilipin 3

```
ATGTCTGCTGATGGTGCTGAAGCTGACGGTAGCACCCAGGTAACCGTTGAAGAACCGGTTCAGCAGCCGTCTGTTGTT
GACCGTGTAGCTTCTATGCCGCTGATCTCTAGCACCTGCGATATGGTGAGCGCGGCGTACGCGTCTACTAAAGAATCT
TACCCGCACATCAAAACCGTTTGCGACGCTGCTGAAAAAGGTGTTCGTACCCTGACCGCTGCTGCTGTTTCTGGTGCG
CAGCCGATCCTGAGCAAGCTGGAACCGCAGATCGCGTCTGCTTCTGAATACGCGCACCGCGGTCTGGACAAACTGGAA
GAAAACCTGCCGATTCTGCAGCAGCCGACCGAAAAAGTGCTGGCTGATACCAAAGAACTGGTTAGCTCCAAAGTTTCT
GGCGCGCAGGAAATGGTTTCTTCCGCTAAAGACACCGTTGCTACCCAGCTGAGCGAAGCGGTAGACGCCACCCGTGGT
GCTGTTCAGTCTGGTGTTGATAAAACCAAATCCGTGGTTACCGGCGGTGTGCAGAGCGTTATGGGCAGCCGTCTGGGT
CAGATGGTTCTGAGCGGTGTTGACACCGTTCTGGGTAAATCTGAAGAATGGGCGGACAACCACCTTCCGCTGACCGAC
GCGGAACTGGCTCGCATCGCGACCTCTCTGGACGGTTTCGACGTTGCGAGCGTTCAGCAGCAGCGTCAGGAACAGTCT
TACTTCGTTCGTCTGGGTAGCCTGTCCGAACGTCTGCGTCAGCACGCTTACGAACACTCTCTGGGTAAACTGCGTGCT
ACCAAACAGCGTGCGCAGGAAGCTCTGCTGCAGCTGTCTCAGGTTCTGTCTCTGATGGAAACCGTTAAACAGGGTGTT
GACCAGAAACTGGTTGAAGGCCAGGAAAAACTGCACCAGATGTGGCTGTCTTGGAACCAGAAACAGCTGCAGGGTCCG
GAAAAAGAACCGCCGAAACCGGAACAGGTTGAATCCCGTGCTCTGACCATGTTCCGTGACATCGCGCAGCAGCTGCAG
GCTACCTGCACCTCCCTGGGTTCCTCTATCCAGGGTCTGCCGACCAACGTTAAAGACCAGGTTCAGCAGGCGCGTCGT
CAGGTTGAGGACCTGCAGGCGACCTTCAGCTCCATCCATTCTTTCCAGGACCTGTCCTCTTCTATCCTGGCTCAGTCC
CGTGAACGTGTAGCGTCTGCGCGTGAAGCGCTGGACCACATGGTTGAATACGTTGCTCAGAACACCCCGGTAACTTGG
CTGGTTGGTCCGTTCGCTCCGGGTATCACTGAAAAAGCTCCTGAAGAGAAAAAATAA
```

Supplementary References

1. Kokorin, A.I. *Nitroxides - Theory, Experiment and Applications*. (IntechOpen, Rijeka; 2012).
2. Rabenstein, M.D. & Shin, Y.K. Determination of the distance between two spin labels attached to a macromolecule. *Proc Natl Acad Sci U S A* **92**, 8239-8243 (1995).
